# Clinical manifestations and anti-TNF alpha therapy of juvenile Behçet’s disease in Taiwan

**DOI:** 10.1186/s12887-019-1613-5

**Published:** 2019-07-11

**Authors:** Ya-Chiao Hu, Yao-Hsu Yang, Yu-Tsan Lin, Li-Chieh Wang, Hsin-Hui Yu, Jyh-Hong Lee, Bor-Luen Chiang

**Affiliations:** Department of Pediatrics, National Taiwan University Hospital, No. 7, Changde Street, Zhongzheng District, Taipei City, Taiwan

**Keywords:** Children, Behçet’s disease, Anti-TNF alpha therapy, Taiwan

## Abstract

**Backgrounds:**

Behçet’s disease (BD) is a rare vasculitic disorder affecting all sizes of vessels. Among BD patients, 4 to 25% of patients with diagnosed age younger than 16 years old are defined as juvenile BD (JBD). This study aimed to evaluate the clinical manifestations and treatments of patients with JBD, with a particular focus on the effectiveness and safety of anti-tumor necrosis factor (TNF)-alpha therapy.

**Methods:**

We retrospectively reviewed data of all patients diagnosed with JBD at age of 16 years or younger in a tertiary hospital in Taiwan. The clinical manifestations, laboratory data, treatments, disease courses, and clinical outcomes were evaluated. The effectiveness of anti-TNF-alpha therapy was measured based on changes in Behçet’s Disease Current Activity Form (BDCAF) scores, prednisolone dosages and the immunosuppression load scores.

**Results:**

Fifty-five patients were included in the study. The median age at disease onset was 11 years. The most common clinical presentation was recurrent oral aphthous ulcers (100%), followed by genital ulceration (69.1%), skin lesions (36.4%), gastrointestinal symptoms (29.1%), ocular involvement (27.3%), and arthralgia (27.3%). Ninety-one percent of the patients fulfilled the International Criteria for Behçet’s Disease, and 36.4% met the Paediatric Behçet’s Disease criteria. The most frequently used medications were prednisolone (74.5%) and colchicine (54.5%). Six patients with refractory or severe JBD received anti-TNF-alpha therapy. These patients were diagnosed at a younger age compared with those who did not receive anti-TNF-alpha therapy (7.5 vs 13 years; *P* = 0.012), the BDCAF scores reduced significantly at the 1st month, the 6th month and 1 year after the treatment. They did not use steroids after the first year of treatment, and, after treatment for 6 months, their immunosuppression load scores reduced significantly. Due to the limited case numbers, literature reviews of anti-TNF-alpha therapy for refractory JBD were conducted, which had a total 18 JBD patients receiving anti-TNF-alpha therapy, of which fifteen patients had favorable outcomes after treatment with minimal side effects.

**Conclusions:**

Anti-TNF-alpha therapy may be necessary for JBD patients with refractory disease courses. Anti-TNF-alpha therapy was effective and safe in these patients, especially regarding its corticosteroid- and immunosuppressive drug-sparing effects.

## Background

Behçet’s disease (BD) is a recurrent, multisystem, inflammatory disorder that affects vessels of all sizes. Its variety of clinical manifestations include recurrent oral and genital aphthous ulcers, skin lesions, arthritis, uveitis, thrombophlebitis, and gastrointestinal and central nervous system involvements [1, 2]. Juvenile BD (JBD) or pediatric BD refers to those patients who was diagnosed with Behçet’s disease at or before the age of 16 [3], and it accounts for 4–25% of all patients with BD [4]. The BD incidence varies according to geographical location, with the highest prevalence in Turkey (420 per 100,000 people), followed by Iran, northern China, and Korea [2]. The estimated BD incidence in Taiwan was 0.9 per 100,000 person-years from 2005 to 2009 [5]. Although the age of peak onset is between the third and fourth decades of life, awareness about the presence of BD during childhood has increased gradually [3, 6, 7]. Given its rarity and variety of clinical presentations, diagnosing JBD is challenging and often delayed.

The pathogenesis of BD remains unclear. While BD is largely considered a type-1 T helper (Th1) and type-17 T helper (Th17)-mediated inflammatory disease, characterized by elevated Th1 and Th17 cytokine levels [2, 8–10], recent evidence suggests that the autoinflammatory nature of BD plays a role in its pathogenesis [11]. Given the complex mechanisms underlying BD, therapy depends largely on the affected site and disease severity. While the European League Against Rheumatism (EULAR) established treatment recommendations for the management of adult BD, no controlled studies have evaluated the treatment of JBD [8]. Immunosuppressive therapy has improved patients’ outcomes, but some BD patients have refractory disease courses. Anti-TNF-alpha therapy is useful for patients with refractory, severe BD, and especially for those with ocular [10, 12], central nervous system [10], and gastrointestinal [9, 10, 13] involvements. The use of anti-TNF-alpha therapy in children has been described in the context of small patient series or case reports only [14–16]. In this study, we evaluated the clinical characteristics of patients with JBD and their treatments, with a particular focus on the efficacy and safety of anti-TNF-alpha therapy.

## Methods

### Study design and enrollment criteria

We retrospectively reviewed patients with diagnosis of BD at age of 16 years or younger at the National Taiwan University Hospital who were diagnosed between 2008 and 2017, and whose diagnostic codes were ICD-9-CM 136.1 or ICD-10-CM M35.2. The patients’ data were derived from their medical records and interviews with the patients and their caregivers. The records contained data describing the patients’ clinical presentations, physical examinations, laboratory data at diagnosis, endoscopic and pathological findings, and medications administered. Patients were recruited if they fulfilled the International Criteria for Behçet’s Disease (ICBD) or the Pediatric Behçet’s Disease (PEDBD) criteria [4]. If patients did not satisfy these criteria, but they had oral or genital aphthous ulcers and some symptoms and signs associated with BD, including arthritis/arthralgia, abdominal pain, gastrointestinal bleeding, or pathological findings, and they did not have a concurrent infection that might contribute to BD-like symptoms, we included them in this study as patients with possible JBD. Those patients who were older than 16 years old at the time of diagnosis or who eventually had another alternative diagnosis, such as inflammatory bowel disease, systemic lupus erythematosus or identified infectious disease with transient BD-like symptoms, were excluded from this study.

The patients were divided into two groups according to the use of anti-TNF-alpha therapy, and the clinical presentations, laboratory data, and other treatment regimens were compared. The therapeutic outcomes of the JBD patients who received anti-TNF-alpha therapy were evaluated at baseline, and at 1 week, 1 month, 3 months, 6 months, 1 year, and > 1 year, if available. The treatment outcomes were evaluated in relation to symptom improvements, inflammatory marker changes, corticosteroid sparing, and the immunosuppression load scores. The Behçet’s Disease Current Activity Form (BDCAF) scores were also collected to evaluate the change of disease severity. The BDCAF total score was calculated out of 12 and then given as a transformed index score on an interval scale out of 20 [17]. The degree of immunosuppression was graded using a semiquantitative scale proposed by Nussenblatt et al [18] and applied to evaluations of anti-TNF therapy by Calvo-Río et al. [12] This grading scheme provides a combined, single numerical score for the total immunosuppression load per unit of body weight per day. Grades for prednisone, cyclosporine, azathioprine, and methotrexate ranged from 0 to 9, and those for mycophenolate mofetil ranged from 0 to 7. For patients administered multiple medications, the sum of the grading score for each drug was used to calculate the total immunosuppression score at baseline, and at 1 week, 1 month, 6 months, 1 year, and 2 years after the anti-TNF-alpha therapy was initiated.

### Statistical analyses

All variables were tested for normality with either Kolmogorov–Smirnov test or Shapiro-Wilk test. Patient data were expressed as counts, percentages or medians with range for quantitative and ordinal data. The statistical analyses were performed using IBM®SPSS® software, version 23.0 for Windows (IBM Corporation, Armonk, NY, USA). Fisher’s exact test was used to compare the categorical variables. Differences between the continuous variables were compared using the nonparametric Mann-Whitney-U test for independent samples or the Wilcoxon signed-rank test for paired related samples. All of the tests were two-sided, and a value of *P* < 0.05 was considered statistically significant.

## Results

### Patients’ characteristics and treatments

Fifty-five patients with JBD were investigated, comprising 22 boys and 33 girls. The median age at the initial onset of symptoms was 11.0 (range 0.1–16.0) years, and the median age at diagnosis was 13.0 (range 0.1–16.0) years. Fifty (90.9%) and 20 (36.4%) patients fulfilled the ICBD and PEDBD criteria, respectively. Five patients had possible JBD. The most common clinical presentation was recurrent oral aphthous ulcers (100%), followed by genital ulceration (69.1%), skin lesions (36.4%), and gastrointestinal symptoms (29.1%) (Table 1). Pathergy test was not routinely performed in our cases. Only 5 patients had records of pathergy test and two of them had positive findings.

**Table 1:** Characteristics of the patients with juvenile Behçet’s disease (*n* = 55)

Characteristic		
Male gender, n (%)	22	(40.0)
Age at disease onset (years)	11	(0.1–16.0)
Age at diagnosis (years)	13	(0.1–16.0)
Time to diagnosis (years)	0	(0.0–8.0)
Diagnosis criteria, n (%)		
ICBD	50	(90.9)
PEDBD	20	(36.4)
No criteria met	5	(9.1)
Clinical presentations, n (%)		
Recurrent oral aphthous ulcers	55	(100)
Genital ulceration	38	(69.1)
Skin involvement	20	(36.4)
Ocular involvement	15	(27.3)
Neurologic signs	2	(3.6)
Vascular signs	1	(1.8)
Gastrointestinal symptoms	16	(29.1)
Arthritis/arthralgia	15	(27.3)

For quantitative and ordinal data median and range are presented

*ICBD* International Criteria for Behçet’s Disease, *PEDBD* Pediatric Behçet’s Disease

The median white blood cell count was 8.8 (range 3.8–18.6) × 10^3^ cells/μL, and 27% of the patients had leukocytosis > 11.0 × 10^3^cells/μL. The median hemoglobin level was 13.0 (range 10.0–14.7) g/L, and 22.7% of the patients had anemia relative to their ages. The median erythrocyte sedimentation rate (ESR) was 21.0 (range 2.0–90.0) mm/h, and 51.5% of the patients had ESRs > 20 mm/h. The median C-reactive protein (CRP) level was 0.6 (range 0–20) mg/dL, and 45.5% of the patients had elevated CRP levels. Antinuclear antibodies were detected in 20.5% of the patients, but no other autoantibodies were detected. Thirteen patients received an examination of HLA typing and only one patient had positive HLA-B51.

The patients most frequently received oral prednisolone (74.5%), followed by colchicine (54.5%) and mesalazine/sulfasalazine (34.5%). Oral prednisolone and colchicine were mostly frequently prescribed as first-line systemic therapy. Nonsteroidal anti-inflammatory drugs were prescribed to 30.9% of the patients and always in combination with other medications. Azathioprine was the most commonly used immunosuppressive drug (30.9%) and the only immunosuppressant used as first-line treatment. Six patients received anti-TNF-alpha therapy. Two patients who presented with uveitis were treated with adalimumab mainly, and the other patients were treated with etanercept (Table 2). After treatment, most of the patients’ symptoms improved, and the initially high CRP levels and ESRs gradually returned to their normal ranges within 6 months. Fifteen patients required long-term follow-up assessments, and, of these, six patients received anti-TNF-alpha therapy and nine patients received colchicine or disease-modifying anti-rheumatic drugs (DMARDs) and, sometimes, prednisolone to manage disease flares.

**Table 2:** Systemic treatments in patients with juvenile Behçet’s disease

	Patients receiving treatment at any time, n (%)	Patients receiving first-line systemic therapy, n (%)
Colchicine	30 (54.5)	24 (43.6)
Oral prednisolone	41 (74.5)	31 (56.4)
NSAIDs	17 (30.9)	–
Mesalazine/Sulfasalazine	19 (34.5)	6 (10.9)
Hydroxychloroquine	12 (21.8)	7 (12.7)
Azathioprine	17 (30.9)	2 (3.6)
Cyclosporine	11 (20.0)	–
Methotrexate	1 (1.8)	–
Mycophenolate mofetil	2 (3.6)	–
Adalimumab	2 (3.6)	–
Etanercept	4 (7.3)	–

*NSAID* nonsteroidal anti-inflammatory drug

### Anti-tumor necrosis factor-alpha therapy for patients with juvenile Behçet’s disease

The six patients who received anti-TNF-alpha therapy were followed continuously for durations that ranged from 1 year to 3 years. Compared with the patients who did not receive anti-TNF-alpha therapy (Table 3), the median ages of those who received anti-TNF-alpha therapy were lower at disease onset (12 vs 7.0 years; *P* = 0.029) and diagnosis (13.0 vs 7.5 years; *P* = 0.012). Compared with the patients who did not receive anti-TNF-alpha therapy, gastrointestinal symptoms (22.4 vs 66.7%; *P* = 0.041) and arthritis/arthralgia (24.5 vs 66.7%; *P* = 0.053) were more frequent in the patients who received anti-TNF-alpha therapy. The groups did not differ regarding other aspects of the clinical presentation of BD. Compared with the patients who did not receive anti-TNF-alpha therapy, those who received anti-TNF-alpha therapy had higher median serum CRP levels at diagnosis (0.4 vs 6.0 mg/dL; *P* = 0.025), and they tended to present with lower median hemoglobin levels at disease onset (13.1 vs 11.5 g/L; *P* = 0.06).

**Table 3:** Comparison of the characteristics of the patients with juvenile Behçet’s disease who were and were not treated with anti-tumor necrosis factor-alpha therapy

Characteristic	Treatment with anti-TNF-α (*n* = 6)		No anti-TNF-α therapy (*n* = 49)		*P* value
Male gender, n (%)	3	(50.0)	19	(38.8)	0.674
Age at disease onset (years)	7.0	(0.25–13.0)	12	(0.1–16.0)	0.029*
Age at diagnosis (years)	7.5	(2.0–13.0)	13	(0.1–16.0)	0.012*
Time to diagnosis (years)	1	(0.0–2.8)	0	(0.0–8.0)	0.553
Clinical presentation, n (%)					
Recurrent oral aphthous ulcers	6	(100.0)	49	(100.0)	1.000
Genital ulceration	5	(83.3)	33	(67.3)	0.654
Skin involvement	2	(33.3)	18	(36.7)	1.000
Ocular involvement	2	(33.3)	13	(26.5)	0.638
Neurologic signs	1	(16.7)	2	(4.1)	0.298
Vascular signs	0	(0.0)	1	(2.0)	1.000
Gastrointestinal symptoms	4	(66.7)	11	(22.4)	0.041*
Arthritis/arthralgia	4	(66.7)	12	(24.5)	0.053
Laboratory data at diagnosis					
WBC count (× 10^3^/ΜL)	11.8	(5.0–18.0)	8.8	(3.8–18.6)	0.339
Hemoglobin, g/L	11.5	(10.3–14.1)	13.1	(10–14.7)	0.060
Platelet count (× 10^3^/ΜL)	386	(212.0–570.0)	294.5	(170.0–576.0)	0.098
C-reaction protein (mg/dL)	6	(2.5–10.5)	0.4	(0.0–20.0)	0.025*
ESR (mm/h)	29	(9.0–50.0)	18.5	(2.0–90.0)	0.314
Medications used at any time					
Prednisolone, n (%)	6	(100)	35	(71.4)	0.320
Colchicine, n (%)	2	(33.3)	28	(57.1)	0.394
NSAIDs, n (%)	3	(50)	14	(28.6)	0.359
DMARDs^‡^, n (%)	4	(66.7)	21	(42.9)	0.394
Immunosuppressive agents^#^, n (%)	6	(100)	15	(30.6)	0.002*
Rheumatologic drugs, n	4	(2–7)	2	(0–6)	0.010*

For quantitative and ordinal data median and range are presented

*TNF* tumor necrosis factor, *WBC* white blood cell, *ESR* erythrocyte sedimentation rate, *NSAID* nonsteroidal anti-inflammatory drug, *DMARD* disease-modifying antirheumatic drug

‡ Included hydroxychloroquine, mesalazine, and sulfasalazine

# Included cyclosporine, mycophenolate mofetil, methotrexate, and azathioprine

*A value of *P* < 0.05 was considered statically significant

We analyzed medications other than anti-TNF-alpha therapy in the two groups of JBD patients. All six patients who received anti-TNF-alpha therapy were treated with long-term oral prednisolone. In the group that did not receive anti-TNF-alpha therapy, 71.4% patients had taken oral prednisolone, and 60% of them had undergone long-term courses of steroid treatment over 14 days. More patients who received anti-TNF-alpha therapy were prescribed immunosuppressants (30.6 vs 100%; *P* = 0.002) and a greater variety of anti-rheumatic medications than those who did not receive anti-TNF-alpha therapy.

**Fig. 1:**
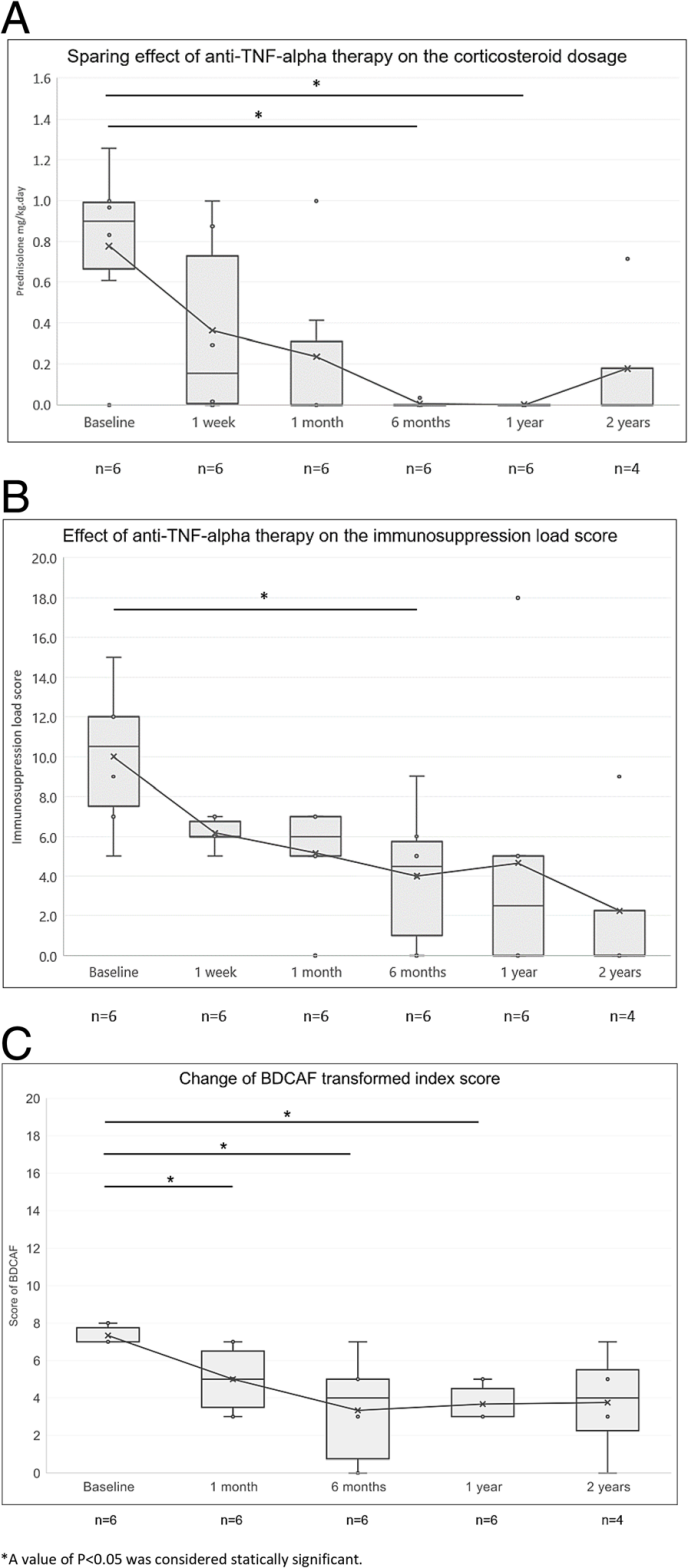
Effect of anti-tumor necrosis factor-alpha therapy in patients with juvenile Behçet’s disease on (**a**) corticosteroid sparing, (**b**) the immunosuppression load score and (**c**) serial change of Behçet’s Disease Current Activity Form transformed index scores. (TNF: tumor necrosis factor; BDCAF: Behçet’s Disease Current Activity Form)

Table 4 summarizes the characteristics of the six JBD patients who received anti-TNF-alpha. Four patients were diagnosed at < 10 years of age, and two patients had experienced symptoms since infancy. All of the patients had received steroids for ≥6 months before the administration of anti-TNF-alpha therapy. Two patients had short statures, and this was considered to be related to chronic disease and long-term steroid use. After starting the anti-TNF-alpha therapy, all six patients’ steroid doses gradually declined over 1 year, and, compared with the baseline dosage, the difference was significant (Fig. 1a). The corticosteroid-sparing effects and reductions in the immunosuppression load scores were observed at 6 months after treatment (Fig. 1a and b). Except for patient #5, all of the patients’ symptoms improved, and their immunosuppressants were discontinued within 1 year. The patients who had high serum CRP levels or ESRs before treatment showed declines in their inflammatory marker levels several months after the anti-TNF-alpha therapy was initiated. Moreover, the serial change of BDCAF scores were depicted in Fig. 1c, which showed significantly reduction at the 1st month, the 6th month and 1 year after the treatment (*P* = 0.042 at 1st month, *P* = 0.027 at the 6th month, and *P* = 0.026 at 1 year).

**Table 4:** Clinical characteristics of juvenile Behçet’s disease patients treated with anti-tumor necrosis factor-alpha therapy and response of the treatment

Case	Sex	Previous treatments	Duration of steroid use before aTNF (years)	Type of aTNF	Duration of aTNF (years)	Reasons for use of aTNF	Treatment responses	Side effects
#1	F	PD, AZA	7.0	ETN	3.2	Frequent fever, oral ulcer, enteritis. Frequent hospitalizations.	Fever subsided and ESR level returned to normal range within 1 year. Steroid was discontinued for 2.8 years. One BD flare after tapering ETN.	Herpes zoster, pneumonia
#2	F	PD, 5-ASA, colchicine	3.3	ETN	2.4	Steroid-dependent disease status with oral ulcers and GI bleeding.	Hgb and CRP level returned to normal range within 4 months. Steroid was discontinued 6 months later. One BD flare after tapering ETN	None
#3	F	PD, AZA Colchicine	1.6	ADA	1.0	Recurrent uveitis, oral and genital ulceration.	Improved uveitis and vessel leakage. VA remained stationary.	None
#4	M	PD, AZA	0.6	ETN	7.8	Refractory oral ulcer, gastrointestinal symptoms.	Improved clinical symptoms; CRP and ESR returned to normal range. PD and AZA were discontinued within 1 year.	Recurrent sinusitis
#5	M	PD, HCQ, CsA, AZA	0.8	ADA	1.4	Steroid-dependent uveitis.	Uveitis subsided with VA improvement. Persistent oral ulcer, high ESR and CRP levels.	None
#6	M	PD, 5-ASA	5.8	ETN	2.7	Neurologic involvement. Steroid dependent disease status with poor drug compliance.	Improved arthralgia, oral ulcer and bloody stool. CRP level returned to normal range. All drugs were discontinued after 6 months.	None

*aTNF* anti-Tumor Necrosis Factor alpha therapy, *PD* prednisolone, *HCQ* hydroxychloroquine, *AZA* azathioprine, *5-ASA* mesalazine, *CsA* cyclosporine, *ETN* Etanercept, *ADA* Adalimumab, *ESR* erythrocyte sedimentation rate, *CRP* C-reactive protein, *Hgb* Hemoglobin, *VA* visual acuity

After receiving adalimumab, the active uveitis and visual acuity of patient #5 improved; however, relapsing oral ulcers, and high CRP levels and ESRs persisted. We discontinued adalimumab and began tocilizumab treatment 1.4 years after the anti-TNF-alpha therapy was initiated, and the patient’s symptoms subsided. When we tried to reduce the anti-TNF-alpha therapy, disease flares occurred in two patients during the second year of therapy. Severe infectious episodes did not occur, and all six patients tolerated the anti-TNF-alpha therapy without experiencing any particular discomfort during treatment.

## Discussion

In this single-center retrospective study, we analyzed patients who were diagnosed with JBD and their treatment. We especially reported the favorable response and safety of using anti-TNF alpha in those patients with refractory symptoms after conventional treatments. Not only the disease severity scores were significantly reduced, but also the dosage of corticosteroid and immunosuppressive drugs were markedly reduced.

The variety of and lack of exact biomarkers for BD has led to the development of several sets of diagnostic criteria for BD. The International Behçet’s Study Group criteria are the most widely used diagnostic criteria for BD, but their sensitivity is suboptimal, especially for JBD [19–21]. Other BD classification criteria have been developed for more robust diagnoses. The ICBD have been validated in JBD patients with sensitivities that range from 70 to 80%, but specificity data are absent [22, 23]. The PEDBD criteria were developed for children, and they have a sensitivity of 73.5% and a specificity of 97.7% [4, 21]. To replicate an actual population of JBD patients, we enrolled JBD patients based on the ICBD, because of their high level of sensitivity for these patients. We also applied the PEDBD criteria to these patients, because they are specific to JBD. In addition to the 50 patients who met either the ICBD or PEDBD criteria, five patients were identified as possible JBD in this study. All of these patients had recurrent oral aphthous ulcers, and they were diagnosed based on their associated symptoms or pathological evidence. They received colchicine or prednisolone, and the symptoms improved in four of these patients. The remaining patient who presented with fever, recurrent oral ulcers, and severe enteritis, experienced persistent symptoms while receiving conventional treatment, and she was eventually administered anti-TNF-alpha therapy. Our study identified pediatric patients who did not fulfill any of the clinical BD criteria, but required therapy like patients with confirmed JBD.

The median age at disease onset and time of diagnosis were 11 and 13 years old respectively in our JBD patients. The mean onset age of JBD reported from other studies [4, 22, 23] ranged from 4.87 to 7.4 years old, which was younger than that of our patients. The reported age at JBD diagnosis was similar amongst those studies (11.29–13.87 years old) and our data. The differences may be explained by the recall bias but it may also be an indication that our physicians were not as alarming as those in other studies in recognizing BD symptoms which appeared in early childhood. There were patients who were diagnosed as BD at an extremely young age in our study group. Among 55 patients, three of them had disease onset at the age less than one-year-old, which were 0.1, 0.25, 0.25 years old respectively. The patient diagnosed as BD at 0.1 years old has been reported as a case report [24]. The case presented with oral ulcer, genital ulcer, and associated GI symptoms, and no evidence of infection were identified after serial studies. She had high serum level of CRP (20 mg/dl) and ESR (90 mm/hr) initially. The symptoms resolved after treatment with short-course of prednisolone and the baby was clinically asymptomatic after 2 months old. There were also other case reports of neonatal BD from Turkey, UK, and Canada [25–28]. Hence despite being rare, diagnosis of BD should be considered in a neonate even if the full diagnostic criteria are not met. The other two cases had symptoms at infancy but fulfilled BD diagnosis at 2 and 3 years old respectively. Both of them had persistent symptoms when they grew up and eventually received anti-TNF alpha therapy.

Specific treatment guidelines for JBD are not available. The EULAR recommends BD treatment should be individualized according to age and gender, the type and severity of organ involvement, and a patient’s preferences [8]. Our data showed that the most frequently used treatments were prednisolone and colchicine, and these comprised first-line therapy for JBD. Compared with the findings from previous studies conducted in other countries, prednisolone was used more frequently in our patients [22, 23, 29]. Among the patients who received oral prednisolone, 24.3% had uveitis and 26.8% presented with gastrointestinal involvements, both of which are associated with poor prognoses [8, 16], and more aggressive treatment was required. Given the rapid responses to systemic corticosteroids and the limited data describing other medications administered to patients with JBD, corticosteroids remain a good choice for short-term therapy.

We found that the patients who received anti-TNF-alpha therapy had used corticosteroids for longer durations and they had used more types of DMARDs and immunosuppressants before they received anti-TNF-alpha therapy. The BDCAF scores were 7 or 8 out of a total score of 20 before they received anti-TNF alpha therapy. Hence, these patients may not have a very severe disease entity but had a refractory disease course even after multiple conventional treatments. Our analysis showed that the patients who received anti-TNF-alpha therapy were younger at disease onset, and two patients were diagnosed with JBD in infancy. The influence of age on JBD has not been described. In addition, the JBD patients who received anti-TNF-alpha therapy had higher serum CRP levels at the time of disease onset and higher rates of gastrointestinal symptoms and arthritis/arthralgia. Intestinal involvement is more frequent in juvenile BD, and its severity can range from mild to hemorrhage or perforation [3]. The disease in patients with intestinal BD sometimes becomes refractory, and other forms of therapeutic intervention and biologics are reported in addition to conventional therapy [14, 15, 30].

TNF-alpha inhibitors treat several rheumatic diseases effectively, even those in children [31, 32]. The findings from several studies that have evaluated the effectiveness and safety of anti-TNF-alpha therapy in adult BD patients with refractory uveitis, have shown symptom improvements in 43.5–67.7% of the patients [10, 12]. Inoue et al reported the treatment response of adalimumab in adult patients with refractory intestinal BD. Sixty percent of the patients showed marked improvements and 20% of them attained complete remission [13]. Among the 6 patients receiving anti-TNF-alpha therapy, we used adalimumab for the two patients with uveitis based on our experience in the treatment of uveitis in juvenile idiopathic arthritis [33, 34]. Furthermore, adalimumab could be reimbursed by the National Health Insurance in Taiwan for patients with uveitis. However, for BD patients with GI tract involvement, adalimumab is an out of pocket expense. Infliximab was not available in Taiwan during the period when our patients had the disease. The other four patients chose etanercept as it was a cheaper choice of anti-TNF alpha agent for these patients. Our results indicated the effectiveness and safety of anti-TNF-alpha therapy in JBD patients whose symptoms were refractory to conventional treatments. Four patients experienced complete symptom remissions and steroid sparing within 6 months. The remaining two patients had partial responses, and their conditions remained stable without disease progression. The reduced BDCAF scores, the changes in the steroid doses and the immunosuppressant load scores after anti-TNF-alpha therapy provided indirect evidence of the patients’ treatment responses, especially after 6–12 months of treatment.

Though several large-scale clinical trials of anti-TNF-alpha therapy in adult BD were conducted, there was a lack of similar trials in pediatric patients. We searched the Medline database published from January 2000 till December 2018 via PubMed. The searching keywords included “Behçet’s disease”, either “child” or “pediatric”, and either “anti-tumor necrosis factor” or “infliximab.” The references which had been cited in other related case report were also reviewed. Literature without full text or in a non-English language was excluded. We found seven case reports and series reported anti-TNF-alpha therapy for refractory JBD [14–16, 35–38]. The details of the reported cases were listed in Table 5. Combining our cases with the previous seven reports of JBD with anti-TNF alpha therapy in literature, there were a total of 18 JBD patients receiving anti-TNF alpha agents. There were more female patients (11/18). The median disease onset age was 10 years old with a range of 2 to 15 years old. The most commonly involved organs in these patients were cutaneous lesions (6/18) and intestinal involvement (6/18), followed by ocular lesions (4/18). Use of adalimumab, infliximab and etanercept have been reported respectively. Ten patients were treated with etanercept, six patients received infliximab, and three of them used adalimumab. Among the 18 cases, fifteen of them had favorable outcomes after treatment, especially in intestinal and ocular lesions, but three patients failed to show any significant improvement, including two cases with Budd-Chiari syndrome. There were some side effects which were mainly non-serious infection. Combining these previous studies and our studies together, using anti-TNF-alpha therapy may be beneficial and safe for refractory JBD patients.

**Table 5:** Review of reported cases with juvenile Behçet’s disease who received anti-tumor necrosis factor alpha therapy in the literature

Case	Sex	Age at diagnosis	Age at aTNF	aTNF	Major organ involvement	Efficacy	Side effect	Reference
1	Female	13	15	IFX	Intestinal lesion	(+)	None	Saulsbury et al. (2003), USA [14]
2	Male	nr	12	IFX	Budd-Chiari syndrome	(−)	None	Seyahi et al. (2007), Turkey [38]
3	Male	15	15	IFX	Budd-Chiari syndrome	(−)	None	
4	Female	13	15	IFX	Ocular lesion	(+)	None	Evereklioglu et al. (2007), Turkey [37]
5	Female	10	11	ETN	Ocular lesion	(+)	Fever	Cantarini et al. (2009), Italy [36]
6	Female	9	13	ETN	Cutaneous lesion	(+)	Bacterial endocarditis	
7	Male	12	14	ETN	Cutaneous lesion	(+)	None	
8	Female	11	13	ETN	Cutaneous lesion	(+)	Fatigue	
9	Male	8	18	IFX	Intestinal lesion	(+)	None	Kaneko et al. (2010), Japan [15]
10	Female	4	9	ETN	Cutaneous lesion	(+)	None	
11	Female	10	12	ADA	Central nervous system	(+)	None	Robinson et al. (2010), USA [35]
12	Female	5	5	IFX	Intestinal lesion	nr^a^	Infusion reaction	Watanabe et al. (2013), Japan [16]
				ETN		(+)	None	

*aTNF* anti-Tumor Necrosis Factor alpha therapy, *ETN* Etanercept, *ADA* Adalimumab, *IFX* Infliximab, *nr* not reported

^a^ IFX in this case was discontinued due to drug infusion reaction

Nevertheless, the anti-TNF-alpha therapy did not cure the JBD patients optimally, as we expected. Two patients in our study experienced disease flares after we reduced the frequency of the anti-TNF-alpha therapy. A previous study reported disease relapses occurred in 58.6% of the adult BD patients 1 year after the anti-TNF-alpha treatments were discontinued [39]. Further investigations are required to determine the timing of treatment cessation. Our group also included a patient who did not reach complete remission until he received tocilizumab, which is a humanized anti-IL-6 receptor monoclonal antibody, 1.4 years after using etanercept. This patient does not use any drugs other than tocilizumab currently. Some publications describe refractory BD patients who received tocilizumab [40–42], but the effectiveness of the treatment varied, and limited numbers of BD patients are described. As evidence of the pathogenesis underlying BD continues to emerge, more therapeutic options become available for severe BD. Large-scale trials that investigate the treatment of BD patients, and especially JBD patients, with biologics are necessary to enable the development of more effective and safe treatments.

This study has some limitations. First, this was a retrospective study, and recall and selection biases existed. Second, a relatively small number of patients received anti-TNF-alpha therapy. Third, not all enrolled cases were evaluated by disease severity score. Nevertheless, this was a relatively large retrospective study of anti-TNF-alpha therapy in Asian JBD patients. Although further larger prospective studies are needed to determine the efficacy and safety of anti-TNF-alpha therapy in JBD patients, it seems that anti-TNF therapy might play an important role for refractory JBD.

## Conclusions

Most of the JBD patients who received corticosteroid- or colchicine-based treatments had favorable outcomes; however, a small proportion of these patients had refractory disease courses even after receiving conventional medication. We found that patients who were younger at disease onset, had higher serum CRP levels at baseline, and presented with gastrointestinal symptoms tended to have poor response to our traditional standard treatments. Anti-TNF-alpha therapy was effective and safe in these patients and it showed corticosteroid- and immunosuppressant-sparing effects.

## Data Availability

The datasets generated and analyzed during the current study are derived from chart review and are not publicly available due to data privacy concerns, but are available from the corresponding author on reasonable request.

## Abbreviations

5-ASA: Mesalazine
ADA: Adalimumab
aTNF: Anti-tumor necrosis factor alpha therapy
AZA: Azathioprine
BD: Behçet’s disease
CRP: C-reactive protein
CsA: Cyclosporine
DMARDs: Disease-modifying anti-rheumatic drugs
ESR: Erythrocyte sedimentation rate
ETN: Etanercept
HCQ: Hydroxychloroquine
ICBD: International Criteria for Behçet’s Disease
JBD: Juvenile Behçet’s Disease
NSAID: Nonsteroidal anti-inflammatory drug
PD: Prednisolone
PEDBD: Pediatric Behçet’s Disease
Th1: Type-1 T helper
Th17: Type-17 T helper
TNF: Tumor necrosis factor
